# Non-pharmacological delirium prevention practices among critical care nurses: a qualitative study

**DOI:** 10.1186/s12912-022-01019-5

**Published:** 2022-08-25

**Authors:** Surui Liang, Janita Pak Chun Chau, Suzanne Hoi Shan Lo, Jie Zhao, Wenhui Liu

**Affiliations:** 1 Nursing Department, Shenzhen Hospital of Southern Medical University, Guangdong, China; 2 Nethersole School of Nursing, The Chinese University of Hong Kong, Hong Kong SAR, China; 3 School of Nursing, Yunnan University of Traditional Chinese Medicine, Kunming, Yunnan China; 4 Nursing Department, Intensive Care Unit, First Affiliated Hospital of Zhengzhou University, Zhengzhou, China

**Keywords:** Delirium, Intensive care units, Non-pharmacological delirium prevention practice, Registered nurses

## Abstract

**Background:**

Delirium is common among critically ill patients, leading to increased mortality, physical dependence, and cognitive impairment. Evidence suggests non-pharmacological delirium prevention practices are effective in preventing delirium. However, only a few studies explore the actual implementation and its associated challenges among critical care nurses.

**Aim:**

To explore critical care nurses’ perceptions of current non-pharmacological delirium prevention practices in adult intensive care settings, including delirium screening, early mobilisation, sleep promotion, family engagement, and sensory stimulation.

**Methods:**

A qualitative design adopting a thematic analysis approach. Semi-structured interviews with 20 critical care nurses were conducted in ten acute hospitals in mainland China.

**Results:**

Three themes emerged: (a) importance of family engagement; (b) influence of organisational factors, and (c) suggestions on implementation. The implementation of non-pharmacological delirium prevention practices was limited by a strict ICU visitation policy, lack of routine delirium screening and delirium training, light and noise disturbances during nighttime hours, frequent resuscitation and new admissions and strict visitation policy. Case-based training, adopting a sensory stimulation protocol, and family engagement may be enablers.

**Conclusion:**

ICU care routine that lacks delirium assessment and the strict family visitation policy made it challenging to implement the complete bundle of non-pharmacological practices. Resource deficiency (understaffing, lack of training) and ICU environment (frequent resuscitation) also limited the implementation of non-pharmacological practices. Clinicians could implement case-based training and sensory-stimulation programs and improve communication with family caregivers by instructing family caregivers to recognise delirium symptoms and delirium prevention strategies.

## Background

Critically ill patients have an increased risk of developing delirium during their intensive care stay. According to DSM-5, delirium is characterised by an acute onset of deficits in attention, awareness, and cognition that fluctuate in severity over time [1, 2]. Delirium affects up to 80% of intensive care unit (ICU) patients [3], and negatively impacts their clinical outcomes (more prolonged ICU and mechanical-ventilation durations, increased mortality, and cognitive impairment), psychological outcomes (depression episodes and post-traumatic stress disorders), and family outcomes (increased anxiety levels and reduced satisfaction of care) [3–6]. Compared with ICU patients without delirium, ICU patients with delirium had 1.9 times longer intubation time (13 vs 6 days) [7] and 1.7 times longer ICU stay (15 vs 9 days) [5], 2.7 times increase in mortality rate (30% vs 11%) [8], 8.2 times increase in long-term cognitive impairment (28% vs 3.4%) [9], and 1.3 times increase in healthcare cost [10]. Fifty-nine percent of family caregivers among ICU patients with delirium showed symptoms of distress, a two-fold increase in anxiety levels, and significantly lower satisfaction levels [11]. Fifty percent of family caregivers are unable to find work and spend at least 50 h a week caring for ICU-discharged patients with delirium [12]. The material cost per ICU delirium patient is about 1,200 euros, about one-third of the total cost during hospitalisation [10].

Non-pharmacological delirium prevention practices may reduce delirium incidence and duration by targeting known risk factors, such as immobilisation, social isolation, sleep deprivation, and sensory deprivation [13]. Delirium prevention is traditionally presented as two different approaches: ‘non- pharmacological’ and ‘pharmacological’. The non-pharmacological approach is, in fact, a multidomain approach, as causal therapies such as the application of antibiotics are subsumed under this concept [14]. A systematic review of 10 randomised controlled trials (RCTs) and 25 non-RCT studies evaluated non-pharmacological delirium prevention practices among critically ill patients to determine the most effective interventions for reducing delirium incidence [15]. Moderate-certainty evidence demonstrated that early mobilisation, family participation, and use of multi-component interventions (e.g., sensory stimulation, delirium screening, sleep promotion, early mobilisation, and family participation) are associated with reduced delirium incidence [15]. Despite the recommended use of single-component or multi-component interventions in clinical practice, a previous multicenter survey found that 30% of ICUs never assessed delirium, and 40% assessed delirium only once a day, regardless of the recommendation by the Society of Critical Care Medicine (SCCM) to optimally screen for delirium three times per day [16]. Meanwhile, among the ICUs that had a delirium screening program, only 42% used validated tools. Furthermore, other practices, such as early mobilisation, sleep promotion, sensory stimulation, and family participation, were only implemented by 31 to 62% of respondents [17]. To conclude, despite the possible significant effects of non-pharmacological delirium prevention practices, the actual implementation rate was low.

Critical nurses, who have continuous contact with patients, are best positioned to screen patients with fluctuations in delirium symptoms and implement early non-pharmacological delirium prevention practices [16]. However, previous qualitative studies mainly focused on investigating nurses’ experiences of caring for delirious patients [18, 19] or delirious patients’experiences in the ICU [20, 21]. Only four studies explored nurses’ perspectives on non-pharmacological delirium prevention as a single component intervention, such as delirium assessment [22], early mobilisation [23, 24], or family engagement [25]. Our study aimed to explore nurses’ perceptions of current non-pharmacological practices for delirium prevention, including delirium screening, early mobilisation, sleep promotion, family participation, and sensory stimulation in adult ICUs. Our results can provide comprehensive insights for promoting the implementation of evidence-based interventions into routine clinical practice.

## Methods

The study was reported in accordance with the consolidated criteria for reporting qualitative studies (COREQ) [26].

### Design

A qualitative approach was utilised. Participants’ experiences and perceptions were elicited by individual, face-to-face discussions with the principal investigator (PI). The PI is a registered nurse in Mainland China and holds a PhD degree. Semi-structured, individual interviews were tape-recorded through an electronic sound card, transcribed verbatim through NVivo, and thematically analysed based on Braun and Clarke’s framework [27], Six steps were adopted in the analyses including becoming familiar with the data, generating initial codes, searching for themes, reviewing themes, defining and naming themes, and producing the report.

### Setting and sample

ICU nurses who have worked in adult ICU settings of tertiary A hospitals for over three years were purposively recruited for the study. Tertiary A hospitals are general hospitals with bed numbers of at least 500 [28]. The ICU settings in Mainland China include medical and surgical ICUs, general ICUs, coronary care units, and respiratory ICUs. A purposive sampling strategy with maximum variation was employed to ensure the greatest variety in terms of participants’ work experience, position, and education to produce a data range for our analysis [29]. We collected and analysed data by following the principle of data saturation, whereby no novel relevant knowledge was obtained from new participants [30].

### Data collection

The data collection lasted from July 2019 to October 2019. A semi-structured interview guide (Table 1) was developed based on a systematic review [31], an integrative review [32], and the input of an expert panel (two nurse academics and two nurse specialists). The PI conducted two pilot interviews to check the readability and appropriateness of the interview guide. The PI, a registered nurse with ICU work experience, followed the interview guide and conducted the semi-structured, face-to-face interviews with participants for 40 min in a quiet meeting room in their working hospitals. The PI, who did not work in the study unit, obtained participant consent. The PI first thoroughly explained the study purposes and procedures, and obtained written informed consent. All interviews were tape-recorded through an electronic sound card [33]. Field notes were taken during and immediately after each interview to record non-verbal expressions for data analysis and interpretation. No participant refused to participate or dropped out during the study.

**Table 1:** Interview guide

Introduction: Thank you for participating in this study. This study aims to use qualitative interviews to understand the current practices of delirium prevention in intensive care units of Mainland China. You can answer those questions freely and ask any questions during the interview. Now let’s begin our discussion.
1. Please tell me your roles and responsibilities of working in the intensive care unit 2. Please tell me about the current practice of preventing and managing delirium in patients at your intensive care unit? 3. What are the current practice of preventing and managing delirium in patients at other intensive care unit in Mainland China, including delirium screening, early mobilisation, sleep promotion, family engagement, and sensory stimulation? 4. From your experiences, how do you find the importance of the practices? 5. How do you find the receipt of these practices by the patients’ family? 6. Which type of practice or strategies do you think is(are) most helpful? 7. What are the challenges in implementing these practices or strategies? 8. What would you suggest the nurses could do in order to improve the prevention and management of delirium in patients at an intensive care unit?
Thank you for all your answers. We will analyse the data from the interview and contact you if additional information is needed!

### Data analysis

The PI transcribed all audiotapes immediately after the interview through NVivo. The accuracy was verified by concurrently reading and listening to the tape-recorded interviews and inviting participants to check their transcripts.

The PI and another investigator independently analysed the data and then met to decide on the best and most appropriate themes in their collective view. First, the two investigators read through all the transcripts word by word to highlight and derive themes. Then, they searched for relevant information on the themes. As this process continued, they further independently reviewed and defined the themes. Both investigators met to discuss and agree on the final themes and sub-themes. Finally, the final themes and sub-themes were sent to the participants, and three participants (2, 3, and 12) provided feedback on the findings, and no further changes were made. All themes and sub-themes are shown in Table 2. Representative quotations from participants were also selected.

**Table 2:** Themes and sub-themes generated

Themes	Sub-themes
(1) Importance of family engagement	A key to sensory stimulation
	A way to support and accompany in care
(2) Influence of organisational factors	Under-staffing
	No established routine for delirium screening
	Frequent resuscitation and new admissions
	Continuous light and noise disturbances
	Strict ICU visitation policy
(3) Suggestions on implementation	Case-based training
	Adoption of a sensory stimulation protocol
	Safety concerns during implementation

### Rigour of the study

This study’s rigour was ensured by adhering to the criteria of dependability, credibility, confirmability, and transferability [34]. The employed strategies included peer debriefing (two members independently analysed the data), an audit trail (detailed record of the decisions made before and during the research and a description of the research process), and a thick description (detailed description of the process, context, and people in the research) [29].

### Ethical consideration

Ethical approval was obtained from the Chinese University of Hong Kong Survey and Behavioural Research Ethics committee (SBRE-18–674). Written informed consent was obtained from all participants by the PI. All five researchers signed confidentiality agreements to ensure participants’ anonymity. Participants’ audiotapes, transcripts, and notes were kept in a secure and encrypted dresser, with access given only to authorised persons [35].

## Findings

We recruited 20 nurses from general (*n* = 7), surgical (*n* = 6), medical (*n* = 3), cardiac (*n* = 2), and respiratory (*n* = 2) ICUs from 10 tertiary-A hospitals in Guangzhou, Zhengzhou, and Hangzhou, Mainland China. The ICU bed numbers ranged from 10 to 45 (Table 3).

**Table 3:** Descriptive characteristics of qualitative study sample (*N* = 20)

Variable	Qualitative Sample (*N* = 20)
**Age (years)**	
25–30	7
31–35	6
36–40	3
> 40	4
**Gender**	
Male	3
Female	17
**Work experience in ICU (years)**	
< 5	6
5–10	8
> 10	6
**Education**	
College	5
Bachelor	11
Master	4
**Title**	
Primary nurse	8
Supervisor nurse	8
Nurse manager	4
**Department**	
General ICU	7
Surgical ICU	6
Medical ICU	3
Respiratory ICU	2
Cardiac ICU	2
**Number of beds**	
10–15	7
16–20	9
> 20	4
**Received Training about ICU delirium**	
Yes	4
No	16

*Note*: *ICU* Intensive care unit

The participants mean age was 33 years (range: 27 − 46 years), and 17 (85%) were female and three were male nurses. Their experience working in ICU ranged from three to 25 years. Educational background levels included college (*n* = 5), bachelor’s (*n* = 11), and master’s (*n* = 4). The study participants were heterogeneous in their demographic characteristics. The four themes and sub-themes were also reflected in similar perspectives **(**Table 3).

### Theme 1: Importance of family involvement

- **(1) A key for sensory stimulation**

Many participants believed that family caregivers could play a critical role in sensory stimulation by talking to patients or providing essential visual or hearing aids.

*“Many ICU patients don’t know whether it’s day or night. And they don’t know what time it is now or where they are. I think it would help if family members were called in to talk to the patients for about 15 min. I think family members should be involved more in the care plan.” (2)*

*“Orientation and sensory stimulation provided by family can benefit patients; for example, patients can be told where they are and about the potential noisy environment of the ICU. We asked family caregivers to buy the patients some magazines or newspapers and download some games for them on an iPad. Although we don’t have specific data, we observed that there were cases of delirium during that period. A standardised sensory stimulation approach may also benefit more patients.” (5)*

- **(2) A way to support and accompany in care**

Some family caregivers support all the care during patients’ ICU stay, while some others might refuse to and even do not want to receive an early mobilisation program due to its high cost.

*“Most family caregivers would like to accompany the patients with delirium whenever needed; however, some family caregivers are reluctant to do so, especially for patients who have stayed in the ICU for a long time.” (4)*

*“Another thing you have to consider is money. Some family caregivers cannot understand why the patients need to receive rehabilitation since they are in a coma, and they think it is a waste of money.” (1)*

### Theme 2: Organisational influence on delirium practices

- **(1) Understaffing**

Participants mentioned that understaffing hindered the implementation of an early mobilisation program in the ICU.

*“One of the obstacles to an early mobilisation program is understaffing. The nurse-to-patient bed ratio in our department is 1:3, and the nurses are very junior. They basically rely on me (the nurse manager) to uphold the standard of practice, and the turnover of nurses is very serious. The daily care routine is always full, and no extra workforce is available to implement an early mobilisation program.” (12)*

- **(2) No established routine for delirium screening**

Participants indicated that delirium assessment was not incorporated into their routine practices and reviewed that their established and traditional routine hindered delirium assessment.

*“Our department has no standard guideline which indicates that we must assess delirium, and we do not use any tool to record it. The doctor mainly prescribes it verbally, so we may not do it very well.” (1)*

Participants reported that they did not receive adequate training on delirium prevention and management and felt confused regarding assessing and managing a patient with delirium. Some expressed difficulty identifying patients with high delirium risks.

*“We previously joined the public training class on delirium through which we learned the definition of delirium and how to assess it. But not every nurse could join it; only those who didn’t go to work could. Now, we can only identify those who had delirium and were obviously combative. The knowledge we learned is insufficient to distinguish those at high risk of delirium.” (6)*

- **(3) Frequent resuscitation and new admissions**

Participants reported that they might need to keep the room lit due to emergencies involving resuscitation and new admissions. Therefore, the sleep quality of patients could be negatively impacted by the noise generated by these activities.

*“Because in ICUs, especially surgical ICUs, if there is a new patient who just had surgery, us nurses would be busy dealing with this patient. I think the light and noise resulting during this process could have a negative impact on other patients.” (19)*

- **(4) Continuous light and noise disturbances**

Almost all participants reported that the room light was consistently turned on throughout the night, though they would turn off a patient’s bed light whenever possible.

*“Basically, I have to work every hour to record the flow and monitor the vital signs, so we rarely turn off the lights.” (7)*

- **(5) Strict ICU visitation policy**

Some participants reported that the visitation duration was limited to 15 to 30 min a day, while others mentioned that no visitation was allowed.

*“Currently, hospital work is very busy, and the 30-min visitation could delay a lot of work and largely disturb our work efficiency.” (11)*

*“In our department, family caregivers are not allowed to come into the ICU. I don’t think it is reasonable since patients may be isolated from their surroundings.” (15)*

### Theme 3: Suggestions on implementation

- **(1) Case-based training**

Delirium training with vivid cases and videos might be beneficial. Although the participants found such case training useful, most of them did not receive case-based training on delirium knowledge.

*“We hope that trainers can record videos of delirious patients’ actions and show them to us during the class because this is more intuitive. If you just tell us how patients with dementia behave or how violent they are, we cannot draw a good enough picture of delirium symptoms.” (3)*

- **(2) Adoption of a sensory stimulation protocol**

Sensory stimulation was one of the strategies proposed by several participants. When assessing the feasibility of sensory stimulation, participants reported positive feedback and believed it was applicable in their ICUs. Compared to other interventions, participants considered a sensory stimulation program the easiest to implement. According to some participants’ observations, such a program may reduce delirium incidence and severity among patients. Participants also mentioned that using a sensory stimulation protocol might improve patients’ clinical outcomes (delirium incidence and severity).

*“Family photographs are presented to the patients. We also ask family caregivers to *regularly* give phone calls or audios to the patients, informing them of the time and the patients’ location.” (2)*

- **(3) Safety concerns during implementation**

Participants mentioned safety concerns during the implementation of non-pharmacological delirium prevention practices, such as management of tubes and the potential infection hazards.

*“Mobilisation, if implemented too early, may induce myocardial infarction or heart failure in patients.” (15)*

*“A critically ill patient tends to have many lines, such as a urinary tube, ventilator tube, and drainage tube. Before and after performing a mobility program, we need to help sort out all the patients’ tubes and devices, which really increases our workload.” (16)*

*“Cross infection is also one of the reasons because many patients have serious infections, and we are worried about their safety.” (15)*

## Discussion

This study explored critical care nurses’ perceptions of non-pharmacological delirium prevention practices. Three themes emerged including importance of family engagement, influence of organisational factors and suggestions on implementation. The implementation of non-pharmacological delirium prevention practices was limited by a strict ICU visitation policy, lack of routine delirium screening and delirium training, safety concerns during implementation, light and noise disturbances during nighttime hours, and frequent resuscitation and new admissions. Case-based training, adopting a sensory stimulation protocol, and family engagement may be enablers.

### Family engagement

Although family engagement is very important for the implementation of non-pharmacological delirium prevention practices, lack of communication between healthcare providers and family caregivers may contribute to the occurrence of uncooperative families. Some family caregivers may not understand why they are not allowed to visit their family members. For those who are allowed to visit the patients, they may not understand why their family members are intubated or sedated. This confusion may even lead them to doubt physicians’ ability to treat their loved ones. Providing more delirium knowledge to family caregivers is therefore vital in enhancing their understanding of nursing care and improving family-caregiver participation [18].

Our study also found that family caregivers sometimes thought rehabilitation was unnecessary to implement and a waste of money. However, a cohort study found that patients in the mobility group had lower hospital costs, fewer delirium days, and improved physical independence compared with those in the usual care group [36]. Hence, extended communication between healthcare providers and family caregivers may help correct caregivers’ misunderstanding about service charges.

### Influence of organisational factors

Adequate staffing plays a critical role in following through with a care bundle [37]. Furthermore, unlike other bundle elements, early mobilisation relies more on teamwork and collaboration [11]. Participants in the present study indicated the importance of a mobility team in the ICU and recommended an implementation team composed of physicians, occupational therapists, nurses, respiratory therapists, nutrition specialists, and family caregivers.

The established, traditional care routine of delirium screening is viewed as a medical responsibility and quality practice standard [38]. Many participants reported that no delirium screening tools were adopted in their departments, which hindered the implementation of delirium screening [39, 40]. Consistently, previous studies reported that 63% of respondents did not employ an ICU delirium-screening tool, and 44% of respondents had never received any training or education on delirium [40]. Therefore, including the recognition of acute delirium as part of the care routine is the key to preventing ICU delirium.

Participants in this study uniformly agreed that patients’ sleep was disturbed due to light, noise, and frequent resuscitation. Light levels in the ICUs might influence melatonin levels, thus altering circadian rhythms [41]. The noise and nursing procedures may cause sleep fragmentation [42]. Physical assessments throughout the evening, night, and early morning hours can also significantly disrupt ICU patients’ sleep patterns and physiology [41]. Innovative strategies for enhancing patients’ sleep quality mentioned by participants included use of earplugs, use of eye masks, and clustering-nursing procedures, the effect of which has been verified by previous studies [43–46].

Our study findings showed a strict visitation policy hindered family engagement, though previous studies reported that family engagement was one of the most important interventions for delirium prevention [47, 48]. Social isolation is a prevalent problem among ICU patients [3], which may be worsened by strict policies, such as limiting telephone and video calls, and restricting the duration of face-to-face visitations to less than an hour. It was reported in a previous multicenter survey that only 538/1521 ICUs were open to family caregivers for 24 h [16]. Thus, hospitals must endeavour to call on influential leaders to review this situation.

### Suggestions on implementation

This study highlights the importance of adopting case-based teaching and learning in delirium training. Case-based learning is a more active approach compared to systematic or passive learning, and has emerged as a popular mode of training [40]. It enhances nurses’ involvement through active participation in daily practices and promotes a change toward patient-centered care [49]. A quasi-experimental study additionally supported that case-based education improved both the utility of the delirium assessment tool, and over time, nurses’ perceptions of the tool’s importance in an ICU setting [50].

Some participants in this study reported the use of sensory stimulation to prevent ICU delirium. They reported using unstructured, incomprehensive, and a variety of methods. A previous study also reported that nurses provided insufficient support to patients who needed glasses/hearing aids, family caregivers did not bring needed items to the ICU, and some patients refused to use them [50]. Indeed, ICUs are not equipped with many communication tools [51]. Some ICUs had a spelling board but only a few patients used it. Lack of time was most often used as a valid excuse for not performing communication through a spelling board or other non-verbal communication methods [22]. However, more and more technological tools and algorithms have been developed to improve communication between healthcare providers, caregivers and patients, including augmentative and alternative communication, and speech enhancement devices [52, 53]. These can replace the time-consuming spelling board, and thus, improve communication.

Despite the lack of structured sensory stimulation, participants in this study widely discussed the benefits of implementing feasible methods, including involving a familiar person, preparing family photographs and records, and employing communication techniques, within the ICU environment. Two pilot RCTs and one feasibility RCT have suggested that a sensory stimulation intervention with family support is highly applicable in preventing and managing delirium in ICUs [46, 47]. One study involved family caregivers in delirium management through seven face-to-face encounters, of which 30 min were dedicated to pre-bedside, and 15 min to bedside and post-bedside phases [54]. In another study, family caregivers were engaged in providing automated reorientation messages to patients over a three-day period in five ICUs [55]. Consistently, providing orientation or memory cues (family photographs, orientation to surroundings) to ICU patients each day is feasible [56]. These studies showed promising results in increasing delirium-free days, reducing delirium severity within patients, and reducing the anxiety levels of family caregivers [54, 55].

### Limitations

Several limitations exist in this study. First, we used a purposive sampling method, which may result in selection bias. We adopted purposive sampling to include various samples with a range of work experience to generate comprehensive understanding. Second, this study relied on a small sample from one country, which may limit the transferability of our findings. However, we selected heterogeneous and representative ICU nurse specialists and head nurses. The third limitation is the lack of interviews with patients and family caregivers, whose perspectives may further enrich our results.

### Implications for practice

Our study highlighted the implementation of non-pharmacological delirium prevention practices in adult ICUs in Mainland China. Some recommendations to improve implementation are suggested. Case-based training is a more active approach than traditional, systematic, or passive learning and could enhance nurse involvement through active participation in daily practices. Practical training would thus help improve ICU nurses’ awareness and capability of delirium assessment. As early mobilisation relies more greatly on teamwork and collaboration [11], a mobilisation team consisting of physicians, occupational therapists, nurses, respiratory therapists, nutrition specialists, and families would benefit from the initiation of an early mobilisation program. Besides, as ICU patients’ sleep quality is adversely impacted due to organisational influence (such as frequent resuscitation and new admissions, continuous light and noise), practices such as eye masks, earplugs, and clustered operations would improve their sleep quality. Furthermore, a lack of communication between healthcare providers and family caregivers may have contributed to the misconception of early mobilisation programs and the uncooperative behaviours of family caregivers. Hence, instructing family caregivers to recognise delirium symptoms and delirium prevention strategies would extend communication between healthcare providers and family caregivers. In addition, family caregivers can also help prepare visual or hearing aids and reorient patients with recordings or family photos. Therefore, such structured sensory stimulation programs with family involvement could contribute to delirium prevention. The safety concerns nurses raised could also be measured as adverse effects in future implementation studies.

## Conclusions

Implementing management strategies to effect changes in ICUs are needed to enhance and sustain the implementation of non-pharmacological delirium prevention practices. Clinicians could incorporate case-based teaching and learning in staff training, implement sensory-stimulation programs, and improve communication with family caregivers by instructing family caregivers to recognise delirium symptoms and delirium prevention strategies.

## Data Availability

The datasets generated and analysed during the current study are available from the corresponding author on reasonable request.

## Abbreviations

ICU: Intensive Care Unit
PI: Principal Investigator
RCT: Randomised Controlled Trial
SCCM: Society of Critical Care Medicine
